# The impact of physical and psychological health outcomes on stigma in people with neuromyelitis optica spectrum disorder: A cross-sectional study

**DOI:** 10.1097/MD.0000000000046738

**Published:** 2025-12-19

**Authors:** Aysa Shaygannejad, Mohammad Yazdan Panah, Saeed Vaheb, Alireza Afshari-Safavi, Vahid Shaygannejad, Omid Mirmosayyeb

**Affiliations:** a Isfahan Neurosciences Research Center, Isfahan University of Medical Sciences, Isfahan, Iran; b Addiction and Behavioral Sciences Research Center, North Khorasan University of Medical Sciences, Bojnurd, Iran; c Department of Biostatistics and Epidemiology, School of Health, North Khorasan University of Medical Sciences, Bojnurd, Iran; d Department of Neurology, Isfahan University of Medical Sciences, Isfahan, Iran.

**Keywords:** cross-sectional, disability, Neuromyelitis optica spectrum disorder, quality-of-life, stigma

## Abstract

Neuromyelitis optica spectrum disorder (NMOSD) is a rare central nervous system autoimmune disease, predominantly marked by optic neuritis and transverse myelitis. In addition to its significant physical burden, NMOSD is associated with psychological challenges, including stigma, which remains underexplored. This study aimed to investigate the stigmatization and its associations with demographic, clinical, and psychological characteristics in people with NMOSD (PwNMOSD). This cross-sectional study recruited 80 PwNMOSD in Isfahan, Iran. Data on demographic, clinical, and psychological features and quality-of-life (QoL) were obtained using medical records, neurological examinations, and standardized instruments. General linear models were employed to assess the relationships between stigma and demographic, clinical, or psychological variables. Stigma was significantly and independently associated with increased disability (Expanded Disability Status Scale, β = 1.34, *P* = .002), impaired mobility (Timed 25-Foot Walk test (T25FW), β = 1.48, *P* < .001), depression (β = 0.21, *P* = .006), and reduced QoL (β = 0.17, *P* < .001). Higher levels of stigma correlated with increased psychological distress and physical impairment. NMOSD-related stigma was strongly linked to disability, depression, and QoL, highlighting the need for targeted interventions. Addressing stigma through psychological and rehabilitative strategies may improve the well-being of PwNMOSD. Future longitudinal studies need to validate these findings.

## 1. Introduction

Neuromyelitis optica spectrum disorder (NMOSD) is a rare and inflammatory autoimmune disease that impacts the central nervous system and is primarily marked by recurrent episodes of optic neuritis (ON) and transverse myelitis (TM), with focal brain inflammation, such as involvement of the brainstem, area postrema, or diencephalic, being less common.^[1–3]^ TM is an uncommon but debilitating inflammatory condition of the spinal cord, arising from diverse etiologies such as immune-mediated, that manifests in heterogeneous clinical patterns.^[4]^ In NMOSD, this inflammation is typically longitudinally extensive, affecting 3 or more contiguous vertebral segments, which helps distinguish it from the more focal or segmental forms of myelitis seen in other disorders like multiple sclerosis (MS).^[5,6]^ NMOSD, previously classified as an MS subtype,^[7]^ is now recognized as a distinct inflammatory demyelinating condition marked by astrocytic damage mediated by aquaporin-4 (AQP-4) antibodies targeting AQP-4-rich regions in the central nervous system.^[7,8]^ MS is a prevalent autoimmune disorder of the central nervous system, in which lymphocytic infiltration induces demyelination and neurodegeneration, commonly causing physical and cognitive deficits in young adults.^[9]^ The NMOSD attacks are characterized by their unpredictable nature and result in the rapid onset of various symptoms, such as limb weakness, numbness, paralysis, eye pain, blindness, pain, impaired bowel and bladder control, extended periods of nausea, vomiting, and hiccups.^[10]^ A considerable number of people with NMOSD (PwNMOSD) exhibit autoantibodies targeting the AQP-4 water channels in astrocytes, leading to secondary demyelination.^[1]^ The current diagnostic criteria classify NMOSD based on the manifestation of primary clinical symptoms (ON and TM), with or without the detection of AQP-4 autoantibodies.^[11]^ Although NMOSD is classified as rare, with a prevalence ranging from approximately 0.5 to 4 per 100,000 individuals, it is significantly associated with substantial disability and reduced quality-of-life (QoL).^[12]^ Due to its rarity and diverse symptomatology, NMOSD can be challenging to diagnose, particularly in the early stages.^[13]^

The infrequent and unpredictable nature of NMOSD imposes a distinct psychological burden on both patients and their families, the precise characteristics and severity of which remain largely undefined.^[14]^ NMOSD attacks significantly diminish health-related QoL (HRQoL) for patients and impose significant financial burdens on the healthcare system.^[15]^ Patients suffering from chronic neurological disorders may encounter considerable stigma, thereby intensifying their disabilities.^[16]^ Stigma is defined as a set of negative and unjustified beliefs or attitudes systematically held by a society or group toward a particular issue.^[17]^ Within healthcare, social stigma involves the rejection or discrimination of individuals or groups based on health conditions that distinguish them from broader societal norms.^[18]^ This can result in the internalization of stigmatizing attitudes, causing individuals to accept the negative views of others, which adversely influences their self-image in comparison to others.^[18]^

Research on the presence of stigma among people with neuroinflammatory and demyelinating disorders, particularly PwNMOSD, is notably limited.^[19]^ Recent investigations into the perception of stigma in people with MS (PwMS) have indicated that stigma is prevalent within this population, being associated with detrimental psychological and physical health outcomes and a decreased QoL.^[17,20–22]^ However, the exploration of stigma and its associated risk factors in PwNMOSD remains poorly addressed in the literature. Notably, a study by Meca-Lallana identified that the prevalence of stigma among PwNMOSD is approximately 60%, which correlates with disability, depression, pain, and fatigue in this population.^[19]^ Furthermore, disease-modifying therapies (DMTs) such as rituximab, a B-cell depleting agent, and azathioprine, an immunosuppressant that inhibits purine synthesis, are commonly used to prevent relapses in PwNMOSD.^[23–26]^ The treatment regimen may influence the clinical stability and psychological outcomes in PwNMOSD.^[27–29]^ Accordingly, this study aimed to explore the stigmatization of PwNMOSD and its association with a broad spectrum of demographic, clinical, and psychological characteristics in this population. This study can advance the limited literature by clarifying the links between stigma, disability, and psychological outcomes in NMOSD. Practically, the findings highlight the need for targeted psychosocial and rehabilitative strategies to reduce stigma and improve quality-of-life in this patient group.

## 2. Methods

### 2.1. Overview of methods

In this cross-sectional study, validated Persian versions of standardized instruments were used to assess physical, psychological, and quality-of-life outcomes. Data were consecutively collected from PwNMOSD in the Isfahan Hakim MS Database through neurological examinations and structured self-report measures, under the supervision of a trained neurologist. Statistical analyses were performed using generalized linear models (GLM) to evaluate the associations between stigma and demographic, clinical, and psychological characteristics.

### 2.2. Ethics

All participants provided written informed consent prior to enrollment. The study protocol and data collection procedures were approved by the Ethics Committee of Isfahan University of Medical Sciences (IR.ARI.MUI.REC.1401.229), and all assessments adhered to the principles of the Declaration of Helsinki.

### 2.3. Participants

Data pertinent to this observational and cross-sectional study were obtained from PwNMOSD, consecutively recruited from the Isfahan Hakim MS Database^[30,31]^ in Isfahan, Iran, between November 2022 and September 2023. The inclusion criteria for the study encompassed patients aged 18 to 65 years, a diagnosis of NMOSD based on the Wingerchuk 2015 criteria^[11]^; being relapse-free or not having received corticosteroid treatment in the past 30 days; and stable treatment for the preceding 3 months. Excluded from the study were patients who experienced difficulties in comprehending or responding to the study questionnaires, as well as those with other chronic disorders that could significantly influence stigma, cognition, or mood. Demographic, clinical, and psychological data were gathered from PwMS, as detailed in the following sections. AQP-4 antibodies were evaluated in PwNMOSD using the cell-based assay technique with indirect immunofluorescence.^[32]^

### 2.4. Outcome measures

An expert neurologist involved in the study collected sociodemographic and clinical data from PwMS while assessing disability, hand function, and gait through the Expanded Disability Status Scale (EDSS),^[33]^ the 9-Hole Peg Test (9-HPT),^[34]^ and the Timed 25-Foot Walk test (T25FW),^[35]^ respectively. Information on DMTs was extracted from medical records. All included patients were on a stable DMT regimen for at least 3 months prior to participation. Moreover, disease duration was calculated as the time (in years) since the initial diagnosis of NMOSD and was included as a continuous variable in the statistical analysis. Each PwNMOSD underwent the following assessment tests and tools. The measurement modalities were divided into 2 categories: Questionnaires assessing self-reported psychological and quality-of-life factors including Beck Depression Inventory-II, BAI, FSS, The MS Impact Scale, Reece Stigma Scale MS, and Performance-based assessments including (EDSS, T25FW test, 9-HPT, Timed Up and Go (TUG), Symbol Digit Modalities Test), which evaluate objective clinical and functional outcomes.

### 2.5. Timed 25-foot-walk (T25FW)

The T25FW is a standardized assessment of ambulatory function, measuring the time an individual takes to walk a distance of 25 feet (7.62 meters) at maximum safe speed. The test was performed using a calibrated digital stopwatch, operated by a trained examiner. Participants completed 2 trials in opposite directions, and the mean completion time (in seconds) was used as the final measure.^[35,36]^

### 2.6. 9-hole peg test (9-HPT)

Upper-limb motor function was evaluated using the 9-HPT, a standardized quantitative measure of finger dexterity. The test was administered with a standard pegboard and 9 plastic pegs, and performance time was recorded using a calibrated digital stopwatch operated by a trained examiner. Participants were instructed to place and remove the 9 pegs, one at a time, into designated holes as quickly as possible. Each participant performed 2 trials with the dominant hand and 2 with the nondominant hand. The average completion time for each hand was calculated and used in the statistical analysis as an indicator of upper-limb motor performance.^[34,37]^

### 2.7. Timed up and go test

The TUG test has been designed to evaluate functional mobility.^[38]^ During the assessment, participants were asked to stand up from an armchair, walk 3 meters at their preferred speed, turn around, walk back to the chair, and sit down. The total time taken to finish the test was recorded in seconds with a stopwatch. Participants were allowed to wear their usual footwear and any foot-assistive devices they typically use. Improvements in mobility on the TUG were reflected by shorter completion times.^[38]^

### 2.8. Reece stigma scale multiple sclerosis

The Persian adaptation of the Reece Stigma Scale for MS (RSS-MS) was employed to evaluate stigma.^[21]^ The RSS-MS comprises 9 items, with responses ranging from “never” to “always” on a 5-point scale, where “never” is scored as one and “always” as 5.^[39]^ The 9 items are as follows: Felt that having MS was a punishment for things I had done in the past; Felt that people were avoiding me because of my MS; Feared that I would lose my friends if they found out about having MS; felt like people that I know were treating me differently because of my MS; felt like people look down on me because I have MS; avoided dating because most people don’t want a relationship with someone with MS; avoided a situation because I was worried about people knowing I have MS; Was embarrassed about having MS; felt that keeping my MS a secret was important. The Persian version of this questionnaire has demonstrated acceptable validity and reliability (Cronbach α = 0.82).^[21]^

### 2.9. The multiple sclerosis impact scale (MSIS-29)

The MS Impact Scale (MSIS-29) is a disease-specific, self-reported measure of HRQoL with high test-retest reliability.^[40]^ It assesses the impact of MS on patients’ QoL over the past 2 weeks. The scale comprises 29 items, divided into 20 physical and 9 psychological components, scored on a 5-point Likert scale (1 = not at all, 5 = extremely). The MSIS-29 comprises 29 items. The physical domain includes 20 items: Do physically demanding tasks? Grip things tightly (e.g., turning on taps)? Carry things? Problems with your balance? Difficulties moving about indoors? Being clumsy? Stiffness? Heavy arms and/or legs? Tremor of your arms or legs? Spasms in your limbs? Your body not doing what you want it to do? Having to depend on others to do things for you? Limitations in your social and leisure activities at home? Being stuck at home more than you would like to be? Difficulties using your hands in everyday tasks? Having to cut down the amount of time you spend on work or other daily activities? Problems using transport (e.g., car, bus, train, taxi, etc)? Taking longer to do things? Difficulty doing things spontaneously (e.g., going out on the spur of the moment)? Needing to go to the toilet urgently? The psychological domain includes 9 items: Feeling unwell? Problems sleeping? Feeling mentally fatigued? Worries related to your MS? Feeling anxious or tense? Feeling irritable, impatient, or short-tempered? Problems concentrating? Lack of confidence? Feeling depressed? Scores range from 29 to 58 (low QoL), 58 to 87 (average QoL), to above 87 (high QoL).^[40]^ The Persian version of MSIS-29, which has a Cronbach alpha of 0.89, was employed in this study.^[41]^

Although RSS-MS and MSIS-29 were originally developed for PwMS, both questionnaires were employed in this study to assess stigma and QoL in PwNMOSD, respectively. This employment is justified by several factors: both instruments have validated Persian versions with high internal consistency^[21,41]^; NMOSD and MS share overlapping clinical manifestations and psychosocial sequelae,^[42,43]^ making these tools relevant for NMOSD populations; and there is currently no NMOSD-specific validated instrument for these constructs.^[19,44]^ Importantly, no modifications were introduced to the original items, and we used the validated Persian versions in their entirety, thereby ensuring conceptual integrity, cross-study comparability, and methodological robustness.

### 2.10. Neuropsychological assessment

The Persian adaptation of the symbol digit modalities test was employed to evaluate cognitive performance in PwNMOSD.^[45]^ This widely recognized test primarily measures information processing speed while also assessing attention, working memory, and incidental learning.^[46,47]^ Participants were provided with a written key associating 9 abstract symbols with corresponding numerical digits, followed by a randomized sequence of symbols with blank spaces for entering the appropriate digits. The performance score, ranging from 0 to 110, represents the total number of correct entries completed within 90 seconds.^[45]^

### 2.11. Beck depression inventory-II

The Beck depression inventory-II (BDI-II), a tool for evaluating behavioral symptoms of depression, was employed in this study in its Persian version, which has established validity and reliability.^[48]^ This 21-item questionnaire evaluates the severity of depressive symptoms across cognitive (e.g., pessimism, worthlessness, indecisiveness), affective (e.g., sadness, loss of pleasure, guilt), and somatic domains (e.g., changes in sleep, appetite, fatigue, and sexual interest), with each item scored on a 4-point scale. Total scores range from 0 to 63, categorized as 0 to 13 (minimal or no symptoms), 14 to 19 (mild), 20 to 28 (moderate), and 29 to 63 (severe depressive symptoms).^[49]^

### 2.12. Beck anxiety inventory

The Persian adaptation of the Beck anxiety inventory (BAI) was employed in this study to assess anxiety.^[50]^ This version of the BAI was previously translated into Persian and validated, demonstrating excellent internal consistency (Cronbach alpha = 0.92) and satisfactory test-retest reliability (*R* = 0.72).^[50]^ The BAI consists of 21 items that measure anxiety symptoms, covering physiological arousal (e.g., heart pounding, dizziness, hot or cold sweats, trembling) and cognitive-affective domains (e.g., fear of dying, fear of losing control, nervousness, and feelings of terror), experienced by participants over the past week. Responses are recorded on a 4-point Likert scale, spanning from 0 (“not at all”) to 3 (“severely, I could barely stand it”). Total scores range from 0 to 63, with higher values reflecting increased levels of anxiety.^[51]^

### 2.13. Fatigue severity scale

The fatigue severity scale (FSS) is a validated 9-item self-report questionnaire used to measure fatigue levels and their impact on motivation, physical activity, and social participation over the past 2 weeks.^[52]^ Items are scored on a 7-point scale, ranging from 1 (“strongly disagree”) to 7 (“strongly agree”), producing a total score between 9 and 63. Higher scores show greater fatigue severity. The cutoff points are defined as follows: scores below 36 represent no-to-mild fatigue, 36 to 52 indicate moderate fatigue, and scores above 52 signify severe fatigue.^[52]^ The Persian version of the FSS has demonstrated high validity and reliability, with a Cronbach alpha of 0.96.^[53]^

### 2.14. Statistical analysis

Data analysis was carried out with IBM SPSS software (Version 25, Armonk). The normality of the data distribution was evaluated utilizing the Kolmogorov-Smirnov test, Q–Q, and P–P plots. Continuous data were expressed as means ± SD or medians with IQR, based on data distribution, while categorical data were presented as frequencies and percentages. GLM were used to examine the relationship between stigma and demographic, clinical, and psychological characteristics. A univariable model was first applied to assess the association between stigma and these characteristics without adjusting for other variables. Subsequently, a multivariable model was constructed, controlling for significant variables identified in the univariable analysis. To address the issue of multicollinearity among depression, anxiety, fatigue, physical MSIS-29, mental MSIS-29, and total MSIS-29, 3 separate multivariable models were developed. Of them, depression, anxiety, and fatigue were entered in Model I. In Model II, physical MSIS-29 and mental MSIS-29 from the aforementioned variables were considered. Ultimately, from those variables, a total of MSIS-29 were entered into Model III. The threshold for statistical significance was established at a *P*-value of .05 or less.

## 3. Results

The study encompassed 80 PwNMOSD, most of whom were women (88.7%, n = 71). Patients with seropositive AQP-4 antibodies comprised 32.5% (n = 26) of the participants. Most PwMS were married (81.2%, n = 65) and occupied (51.3%, n = 41). The mean age was 36.5 years (SD = 7.5), with an average disease duration of 5.7 years (SD = 3.4). The median (IQR) EDSS of PwNMOSD was 2.5 (2). Table 1 provides a detailed description of the demographic, clinical, and psychological characteristics of PwNMOSD.

**Table 1:** Demographic and Clinical Characteristics of People with NMOSD (N = 80).

		Count (%)	Mean (SD)	Median (IQR)
Age	–	–	36.5 (7.5)	36 (9)
Sex	Male	9 (11.3)	–	–
	Female	71 (88.7)	–	–
Marital status	Single	13 (16.3)	–	–
	Married	65 (81.2)	–	–
	Divorced	2 (2.5)	–	–
Employment status	Unoccupied	39 (48.7)	–	–
	Occupied	41 (51.3)	–	–
Education (year)	–	–	14.7 (2.8)	16 (4)
AQP-4 Antibodies	Seronegative	54 (67.5)	–	–
	Seropositive	26 (32.5)	–	–
DMTs	Azathioprine	6 (7.5)	–	–
	Rituximab	74 (92.5)	–	–
Disease Duration (year)	–	–	5.7 (3.5)	5 (4.5)
Age at Disease Onset	–	–	30.77 (7.6)	30 (9.9)
EDSS	–	–	2.52 (1.2)	2.5 (2)

AQP-4 = aquaporin-4, DMTs = disease-modifying therapies, EDSS = expanded disability status scale, NMOSD = neuromyelitis optica spectrum disorder.

### 3.1. Association between stigma and demographic, clinical, psychological, and quality-of-life among PwNMOSD

Univariable GLM indicated that stigma was directly associated with unemployment (β = 3.23, *P* = .035), EDSS (β = 1.65, *P* = .008), T25FW (β = 2.8, *P* < .001), depression (β = 0.35, *P*-value < 0.001), anxiety (β = 0.4, *P* < .001), fatigue (β = 0.22, *P* < .001), physical MSIS-19 (β = 0.28, *P* < .001), mental MSIS-29 (β = 0.45, *P* < .001), and total MSIS-29 scores (β = 0.18, *P* < .001), while inversely associated with age (β = −0.28, *P* = .005) and age at disease onset (β = −0.26, *P* = .009) (Table 2).

**Table 2:** The relationship between stigma and demographic, clinical, and psychological characteristics of people with NMOSD.

		Univariable		Multivariable					
				Model I		Model II		Model III	
		B (S.E.)	*P*-value	B (S.E.)	*P*-value	B (S.E.)	*P*-value	B (S.E.)	*P*-value
Age	−	−0.28 (0.1)	**.005**	−0.15 (0.15)	.298	−0.1 (0.13)	.445	−0.1 (0.13)	.460
Sex (Ref. = Female)	−	−3.27 (2.46)	.185	−	−	−	−	−	−
Marital status (Ref. = Single)	Married	−2.31 (2.1)	.272	−	−	−	−	−	−
	Divorced	4.73 (5.25)	.368	−	−	−	−	−	−
Employment status (Ref. = Occupied)	−	3.23 (1.53)	**.035**	1.15 (1.01)	.258	0.11 (0.92)	.905	0.09 (0.91)	.922
Education	−	−0.19 (0.28)	.499	−	−	−	−	−	−
DMTs (Ref. = rituximab)	−	−3.8 (2.95)	.199	−	−	−	−	−	−
Disease duration	−	−0.06 (0.23)	.806	−	−	−	−	−	−
Age at disease onset	−	−0.26 (0.09)	**.009**	0.03 (0.14)	.810	-0.04 (0.13)	.753	-0.05 (0.13)	.716
EDSS	−	1.65 (0.62)	**.008**	**1.04 (0.49**)	**.034**	**1.34 (0.42**)	**.002**	**1.34 (0.42**)	**.002**
TUG	−	0.54 (0.31)	.089						
T25FW	−	2.8 (0.41)	**<.001**	**1.82 (0.36**)	**<.001**	**1.46 (0.33**)	**<.001**	**1.48 (0.33**)	**<.001**
SDMT	−	−0.12 (0.09)	.239	−	−	−	−	−	−
Depression	−	0.35 (0.04)	**<.001**	**0.21 (0.07**)	**.006**	−	−	−	−
Anxiety	−	0.4 (0.05)	**<.001**	0.09 (0.09)	.271	−	−	−	−
Fatigue	−	0.22 (0.05)	**<.001**	0.001 (0.04)	.993	−	−	−	−
MSIS-29-Physical	−	0.28 (0.03)	**<.001**	−	−	**0.19 (0.05**)	**<.001**	−	−
MSIS-29-Mental	−	0.45 (0.05)	**<.001**	−	−	0.13 (0.07)	.084	−	−
MSIS-29-Total	−	0.18 (0.02)	**<.001**	−	−	−	−	**0.17 (0.02**)	**<.001**

Significant *P*-values are shown in **bold**.

Model I: RSS versus BDI, BAI and FSS.

Model II: RSS versus MSNQ-Physical and MSNQ-Mental.

Model III: RSS versus MSNQ-Total.

DMTs = disease-modifying therapies, EDSS = expanded disability status scale, MSIS-29 = multiple sclerosis impact scale, NMOSD = neuromyelitis optica spectrum disorder, SDMT = symbol digit modalities test, T25FW = timed 25-foot walk test, TUG = timed up and go.

Subsequently, these variables were entered into multivariable GLM analyses to identify independent relationships. In Model I, the association between stigma and age, employment status, age at disease onset, EDSS, T25FW, depression, anxiety, and fatigue, using the “Enter” method, was examined. The results showed that EDSS (β = 1.04, *P* = .034), T25FW (β = 1.82, *P* < .001), and depression (β = 0.21, *P* = .006) were significantly associated with stigma in PwNMOSD (Table 2).

Model II assessed the association of stigma with age, employment status, age at disease onset, EDSS, T25FW, physical MSIS-29, and mental MSIS-29 using the “Enter” method, revealing that stigma in PwNMOSD was significantly linked to EDSS (β = 1.34, *P* = .002), T25FW (β = 1.46, *P* < .001), and physical MSIS-29 (β = 0.19, *P* < .001) (Table 2).

Model III explored the association between stigma and age, employment status, age at disease onset, EDSS, T25FW, and total MSIS-29 using the “Enter” method, identifying EDSS (β = 1.34, *P* = .002), T25FW (β = 1.48, *P* < .001), and total MSIS-29 (β = 0.17, *P* < .001) as significant factors related to stigma in PwNMOSD (Table 2).

## 4. Discussion

This study aimed to explore the impact of physical and mental health on the stigma experienced by PwNMOSD. This study investigated the potential relationships between stigma and a broad spectrum of demographic, clinical, psychological, and QoL factors among PwNMOSD. The findings of this study revealed that EDSS, T25FW, depression, and QoL, particularly its physical components, were independently associated with stigma in PwNMOSD.

Our findings extend NMOSD evidence by showing that stigma aligns closely with both disability and mood-related burden. In NMOSD, multicenter evidence demonstrates that perceived stigma is frequent and closely linked to disability, depressive symptoms, pain, fatigue, and decreased HRQoL.^[19]^ Patient-reported data show that fatigue, pain, and depression consistently impair HRQoL, including occupational functioning, irrespective of relapse activity.^[15]^ Large AQP-4-IgG–positive cohorts further indicate that walking impairment and pain predominantly account for physical HRQoL variance, whereas depression explains mental HRQoL variability.^[54]^ This work advances understanding of stigma in PwNMOSD by applying a multivariable model encompassing both clinician-rated disability and patient-reported indices of depression, fatigue, and HRQoL. In contrast to earlier NMOSD studies based on bivariate associations,^[15,19]^ this analysis employed separate models to account for collinearity among mood and HRQoL domains, thereby isolating the distinct contributions of mobility and psychological factors to stigma and yielding conservative effect estimates within a unified framework.

The perception of stigma among NMOSD patients has been minimally investigated using standardized measures. PwNMOSD experience psychological and physical consequences that are primarily influenced by the severity of their symptoms.^[15]^ Uncertainty surrounding the disease trajectory of NMOSD has been associated with fears of disability and reduced capacity for performing activities of daily living.^[55,56]^ Stigma has a strong and negative relationship with physical and psychological QoL in PwNMOSD.^[19]^

Among PwNMOSD, physical difficulties have a significant, moderate-to-low correlation with stigma.^[15]^ Nearly all PwNMOSD reported some degree of impairment in achieving goals or participating in work and other activities, indicating that work-related functional impairment may be a significant concern for this population.^[15,57]^ PwNMOSD with visible symptoms of NMOSD, such as vision loss or mobility impairment, reported that stigma associated with disability adversely affected their relationships with friends and family.^[14]^ Walking impairment, along with pain severity, serves as an independent predictor of variability in the physical QoL of PwNMOSD.^[54]^ Although NMOSD and MS are distinct clinical entities, they share some symptomatic and psychosocial features, such as chronic disability.^[58,59]^ Since evidence on the link between disability and stigma in NMOSD is limited, findings in MS may offer insights. The extent of disability is most significantly associated with the severity of stigma experienced by PwMS.^[20]^ PwMS who report poorer self-rated health, greater disability, or have progressive MS are more prone to experiencing stigma.^[60,61]^ Self-reported disability levels in PwMS are associated with increased stigma,^[62,63]^ while the relationship between objectively measured disability and stigma remains inconsistent.^[64,65]^ This underscores the importance of incorporating subjective experiences alongside objective health assessments.^[17]^

Stigma has a profound negative impact on individuals with chronic illnesses and is associated with higher levels of depressive symptoms.^[66]^ Invisible symptoms, such as depression, may also contribute to stigma in PwNMOSD. Limited data are available on psychiatric comorbidities in PwNMOSD. According to a recent systematic review and meta-analysis, 40% of PwNMOSD experience depression.^[67]^ Although specific triggers for depression are not yet documented in the literature, PwNMOSD have reported grief following diagnosis, depression associated with departures from values-based living, and stigma resulting from the misunderstood and often “invisible” nature of the disorder.^[14]^ In MS, some depression symptoms may influence perceptions of stigma, but evidence for this association in NMOSD is insufficient. PwMS experiencing low motivation from depression may face a higher risk of isolation, which can be interpreted as a sign of stigma.^[68]^ Mood symptoms were identified as mediators in the relationship between stigma and QoL among PwMS.^[22]^ Depression is a predictor of reduced QoL,^[69]^ potentially explaining the link between stigma and QoL in PwMS.^[17]^ Patients with rare diseases experience a lower QoL compared to both the general population and patients with common chronic illnesses.^[70]^ Examining the interaction between mood symptoms, QoL, and stigma enables the design of psychological interventions for PwNMOSD that are accessible and effective in delivering relief. To address potential multicollinearity between mood symptoms and QoL, they were analyzed in separate multivariable models to mitigate this issue and identify independent associations with stigma.

Although NMOSD and MS are both autoimmune demyelinating diseases of the central nervous system, they differ significantly in terms of immunopathogenesis, clinical course, and therapeutic response.^[11,12]^ NMOSD is primarily associated with antibodies against AQP-4, whereas MS involves T-cell-mediated autoimmune responses with heterogeneous immunological mechanisms.^[11,71]^ The course of MS is typically chronic and progressive, while NMOSD often presents with severe attacks followed by partial recovery, leading to stepwise accumulation of disability.^[12,55]^ Despite these differences, some MS-specific assessment tools, like the EDSS, are frequently used in NMOSD research due to the lack of NMOSD-specific validated instruments.^[72,73]^ Recent studies have emphasized the importance of developing disease-specific outcome measures for NMOSD to reflect its clinical manifestations and progression more accurately. For instance, due to the absence of a stigma assessment tool specific to NMOSD, instruments designed for MS are currently employed. These channelings underscore the need for developing and implementing NMOSD-specific clinical and psychological assessment tools.

Given the significant association between stigma, depression, and reduced QoL in PwNMOSD,^[22]^ psychological interventions targeting these domains may be warranted. Due to the limited evidence specific to NMOSD in this area, we have drawn on findings from other neurologic disorders with overlapping psychological profiles, such as MS, to understand better and propose relevant intervention strategies. Cognitive-behavioral therapy has shown efficacy in reducing internalized depressive symptoms (may potentially reduce stigma) in people with chronic illness.^[74,75]^ Additionally, psychoeducational programs can improve illness understanding, promote adaptive coping, and reduce social withdrawal.^[76]^ Group-based stigma-reduction interventions and acceptance and commitment therapy have also demonstrated benefits in reducing psychological distress and enhancing quality-of-life in MS,^[77,78]^ and may be adaptable for the NMOSD population. Importantly, such interventions should be customized to the specific needs of PwNMOSD. Integrating mental health support into standard NMOSD care may improve patient outcomes beyond clinical disease control.^[79]^

Conceptually, positioning stigma at the interface of clinician-rated disability and patient-reported symptoms clarifies its co-variation with functional limitations beyond mood states.^[19,54]^ NMOSD research highlights the high prevalence and clinical relevance of depression, pain, fatigue, and occupational impairment,^[15]^ supporting the integration of structured psychosocial interventions into standard care. Targeted programs addressing mobility, pain, and early detection of depression may reduce the adverse effects of stigma on functioning and well-being in PwNMOSD.

## 5. Limitations

This study inherits some limitations that should be considered when interpreting its findings. The cross-sectional nature of the current study restricted the ability to evaluate the directionality of relationships between stigma and other variables. Although efforts were made to recruit participants from diverse racial and ethnic backgrounds, the majority of the sample consisted of Caucasian individuals, despite NMOSD being more common in nonwhite populations.^[80]^ Given that PwNMOSD experienced symptoms, such as motor limitations and fatigue, which may hinder their ability to complete questionnaires, this may potentially lead to an underestimation of our results. Moreover, our study did not assess vision-specific outcomes, such as visual impairment due to ON, which may have contributed to stigma among PwMS and limited the scope of our findings. Furthermore, this study employed clinical and psychological assessment tools originally developed for MS, such as RSS-MS and MSIS-29, which may not fully capture the specific symptomatology and disease burden of NMOSD. This highlights the need for developing NMOSD-specific outcome measures. More longitudinal studies are needed to validate our findings. Additionally, the relatively small number of participants for certain assessments and the absence of random sampling at the national or regional level may limit the generalizability of our findings. Nonetheless, consecutive recruitment from a major referral center strengthens the reliability of the collected data. Furthermore, although the Persian version of the FSS has shown excellent internal consistency (Cronbach alpha = 0.96) in 50 PwMS and 30 healthy adults,^[53]^ such a high reliability coefficient may partly reflect item overlap. Therefore, further validation of this instrument in larger samples of NMOSD is warranted. More longitudinal studies are also needed to validate our findings. To the best of our knowledge, this is the first study that assessed the potential relationship between stigma and various factors such as demographic characteristics, education, DMTs, disease duration, different measures of disability, cognitive function, depression, anxiety, fatigue, and QoL among PwNMOSD.

## 6. Conclusion

The findings of this study demonstrated an independent relationship between stigma and a range of adverse psychological and physical health outcomes in PwNMOSD, including increased disability, depression, and reduced mental and physical QoL. As the psychological impacts of NMOSD are frequently neglected, this study underscores the necessity of healthcare professionals to identify patient profiles most susceptible to stigma to deliver targeted interventions addressing stigma and its associated risks. Longitudinal and comprehensive studies are needed to explore stigma vulnerability, resilience, and their associated factors among PwNMOSD.

## Author contributions

**Conceptualization:** Mohammad Yazdan Panah, Vahid Shaygannejad, Omid Mirmosayyeb.

**Data curation:** Aysa Shaygannejad, Saeed Vaheb.

**Formal analysis:** Alireza Afshari-Safavi.

**Investigation:** Mohammad Yazdan Panah, Vahid Shaygannejad.

**Methodology:** Mohammad Yazdan Panah, Omid Mirmosayyeb.

**Project administration:** Omid Mirmosayyeb.

**Resources:** Vahid Shaygannejad.

**Supervision:** Omid Mirmosayyeb.

**Validation:** Omid Mirmosayyeb.

**Visualization:** Mohammad Yazdan Panah.

**Writing – original draft:** Mohammad Yazdan Panah.

**Writing – review & editing:** Vahid Shaygannejad, Omid Mirmosayyeb.
